# A systematic review of semaglutide-associated kidney injury case reports

**DOI:** 10.3389/fmed.2026.1793349

**Published:** 2026-06-01

**Authors:** Xian Xiu, Pan-pan Zheng, Yang Li, Meng Du, Xi Min, Xiaohua Pang, Lili Zhang, Jingtao Liu, Zhe Zhao, Zan-chao Liu, Cai-Xia Feng

**Affiliations:** 1 Hebei Key Laboratory of Basic Medicine for Diabetes, The Second Hospital of Shijiazhuang, Shijiazhuang, Hebei, China; 2 Changjiang Community Health Service Center, High-Tech Industrial Development Zone, Shijiazhuang, Hebei, China; 3 The Fifth Hospital of Shijiazhuang, Shijiazhuang, China

**Keywords:** adverse drug reaction, case, kidney injury, literature analysis, semaglutide

## Abstract

**Background:**

Although semaglutide is widely used due to its efficacy in glycemic control and weight reduction, cases of the associated adverse drug reactions (ADRs), including kidney injury, have gradually increased. This systematic review aimed to summarize reported cases and case series of semaglutide-associated renal injury and their clinical and laboratory features.

**Methods:**

Case reports and case series on ADRs associated with semaglutide published in English and Chinese were systematically retrieved using relevant keywords from PubMed/MEDLINE, Web of Science, Embase, the Cochrane Library, and the Chinese National Knowledge Infrastructure (CNKI) database from database inception to October 31, 2025. Demographic characteristics, clinical information, ADRs, kidney-related laboratory tests, and outcomes were extracted. We used the Joanna Briggs Institute Critical Appraisal Checklist for Case Reports (JBI Case Report Checklist) to evaluate the quality of each included study.

**Results:**

A total of 20 cases (7 males and 13 females) from 18 studies were included after screening 1039 selected publications, with patient ages ranging from 29 to 83 years. Most patients received semaglutide via subcutaneous injection for weight loss or blood sugar reduction. Except for one case with unknown history, all patients had a pre-existing medical condition and were receiving concomitant medications. ADRs occurred at the standard recommended dose, ranging from 0.25 to 2.0 mg and included acute kidney injury (AKI), acute renal failure (ARF), and acute interstitial nephritis (AIN). Twelve cases were diagnosed through renal biopsy, and the remaining eight cases were diagnosed based on renal function parameters. The dosage varied from 0.25 to 2.0 mg per week. The majority of patients developed ADRs after multiple administrations. The clinical manifestations of the patients were diverse. Following treatment, 17 patients improved or recovered, outcomes were not reported in two patients, and one patient did not recover.

**Conclusion:**

Semaglutide use may be associated with kidney damage, which may be irreversible. Clinicians should be cautious when prescribing semaglutide to individuals with underlying conditions and those receiving concomitant medications, and should ensure regular monitoring of kidney function after treatment initiation.

## 1 Introduction

Glucagon-like peptide-1 (GLP-1), a hormone derived from the intestine, is released after eating and stimulates insulin secretion from the pancreas in a glucose-dependent manner (1). Glucagon-like peptide-1 receptor agonists (GLP-1 RAs) are synthetic analogues of endogenous GLP-1, which can bind to and activate GLP-1 receptors. Compared with native GLP-1, these agents exhibit improved pharmacokinetic properties and can achieve stable and longer-lasting therapeutic effects (2, 3). GLP-1 RAs achieve better blood sugar control through multiple mechanisms, including stimulation of blood sugar-dependent insulin secretion, inhibition of glucagon secretion, delayed gastric emptying, and reduction of calorie intake. GLP-1 RAs can bind to GLP-1 receptors that are widely distributed in various tissues, including the pancreas, kidneys, heart, brain, and gastrointestinal tract and exert effects through modulation of the incretin axis. However, GLP-1 RAs exhibit significant differences in molecular size and structure, chemical and physiological properties, and receptor affinity, which contribute to variability in their pharmacodynamic profiles and clinical outcomes (3, 4).

With the alarming increase in the prevalence of type 2 diabetes (T2D) and its associated complications, the need for effective therapeutic strategies have become increasingly urgent (5). Randomized controlled trials have demonstrated that GLP-1RAs are more effective than conventional hypoglycemic drugs in improving blood sugar control and promoting weight loss (6). Since 2005, the US Food and Drug Administration (FDA) has approved several GLP-1 Ras, including liraglutide, dulaglutide, exenatide, and lixisenatide, for the treatment of T2D. In 2017 and 2019, the FDA approved Ozempic and Rybelsus (oral semaglutide), respectively, for the treatment of T2D. Therefore, semaglutide is currently the only GLP-1 AR available in the market in both subcutaneous and oral formulations (7, 8). Moreover, due to its longer half-life, it can be injected once a week. Furthermore, semaglutide, in addition to its potent glucose-lowering effects, exhibit remarkable effects such as sustained weight reduction and metabolic benefits. It has also been reported to promote physiological effects such as reducing cardiovascular risks, preventing atherosclerosis, and providing renoprotective and neuroprotective effects (9–13). These effects have contributed to its widespread clinical adoption. Although semaglutide has a favorable safety profile (14), apart from the potential benefits of developing new hypoglycaemic drugs, regulatory agencies and clinicians are increasingly concerned regarding its long-term safety.

Studies have shown gastrointestinal disturbances as the most common adverse drug reactions (ADR) associated with semaglutide. However, the risk of kidney disease should not be overlooked (5, 15). Pharmacovigilance analysis using public databases, such as the FDA Adverse Event Reporting System (FAERS) have identified ADR signals suggesting an association between kidney injury and semaglutide (15). Although many systematic reviews on the safety of semaglutide have been published in recent years (16–18), comprehensive comparative studies on semaglutide-induced kidney damage in adults remain limited. Therefore, the present study aims to collect and analyze case studies related to semaglutide -induced kidney damage, providing a more comprehensive theoretical basis for the rational use of semaglutide in adults with or without diabetes mellitus.

## 2 Methods

This systematic review of case reports was conducted and reported following the Preferred Reporting Items for Systematic Reviews and Meta-Analyses (PRISMA) statement (19).

### 2.1 Search strategies

A comprehensive literature search was conducted using the PubMed/MEDLINE, Web of Science, Embase, the Cochrane Library, and CNKI databases to identify relevant case reports and case series published in English or Chinese from database inception to October 31, 2025. The search strategy included the following terms: (“semaglutide” [Title/Abstract]) AND (“case report” OR “case presentation” OR “case summary” OR “case description” OR “case discussion”). Supplementary Table 1 provides the detailed information on the database search. To obtain comprehensive literature, relevant review articles were also screened, and the reference lists of included studies were manually searched to identify any additional relevant articles.

### 2.2 Inclusion and exclusion criteria

The inclusion criterion was original case reports describing kidney injury associated with semaglutide use. The exclusion criteria were as follows: (i) review articles, meta-analyses, study protocols, books, letters, and others; (ii) reports describing non-renal ADRs related to semaglutide; (iii) studies not involving semaglutide, ADRs, ADRs associated with semaglutide, and case reports; (iv) duplicate reports derived from the same case; (v) Seriously lacking of the case data.

### 2.3 Study selection and data extraction

After literature retrieval, two investigators (XX and CXF) independently screened the titles, abstracts, and full texts according to the inclusion and exclusion criteria. A third reviewer (PPZ) conducted a comprehensive review of the selected articles to confirm their eligibility for inclusion. After final selection, data were extracted by three investigators (XX, PPZ, and CXF) from the eligible case reports, which included first author’s name, year of publication, country, patient sex and age, medical history, indication for medication, route of administration, dosage, concomitant medications, ADRs, diagnosis, time to ADR onset, clinical presentation, drug re-exposure, intervention measures, laboratory parameters, such as blood urea nitrogen (BUN), serum creatinine (SCr), estimated glomerular filtration rate (eGFR), urine protein-to-creatinine ratio (UPCR), and proteinuria, and patient outcomes. Discrepancies in the collected data were discussed among the three investigators (XX, PPZ, and CXF) and resolved by consensus. The extracted data were repeatedly verified three times.

### 2.4 Quality assessment

The quality assessment of the included studies was performed according to the JBI Case Report Checklist (20). Two investigators (XX and CXF) independently assessed each report based on the following criteria: patient demographic characteristics, patient medical history, clinical condition of patient at presentation, diagnostic tests or assessment methods and their results, interventions or treatment procedures, post-intervention clinical condition, identification of adverse events, and key takeaways.

## 3 Results

### 3.1 Search results

**FIGURE 1:**
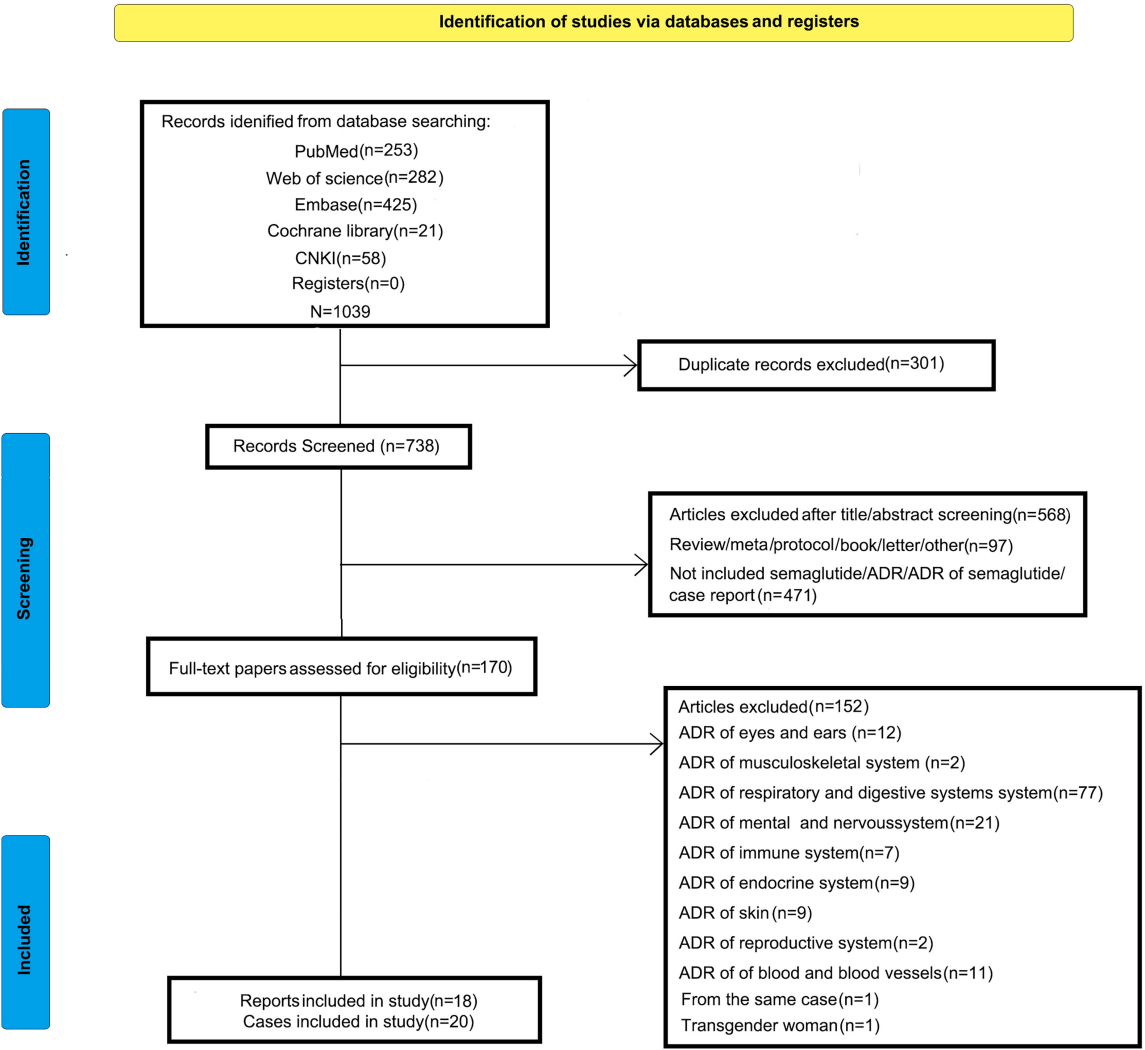
Flowchart of literature screening for this systematic review.

The comprehensive literature search identified a total of 1,039 potentially relevant articles, including 253 from PubMed, 282 from Web of Science, 425 from Embase, 21 from the Cochrane Library, and 58 from CNKI. After removing duplicates and screening titles and abstracts, 170 studies were assessed for full-text eligibility. Ultimately, 18 studies (15, 21–36) met the inclusion and exclusion criteria and were included in this systematic review. The literature retrieval process is shown in Figure 1.

### 3.2 General information

A total of 20 cases from five countries were included, including 7 males and 13 females, with ages ranging from 29 to 83 years. Except for one unknown case, all 19 cases had a previous medical history, among which 13 patients had T2D, 12 had hypertension, and 7 had chronic kidney disease (CKD). Semaglutide was primarily prescribed for T2D, and weight loss was also reported as an indication in eight cases. The predominant route of administration was subcutaneous injection. Unless otherwise specified in the original reports, all patients had a history of concomitant medications. Table 1 summarizes the baseline characteristics of the included studies.

**TABLE 1:** The basic information of the 20 included cases.

References	Region	Gender (F/M)	Age	Past medical history	Reasons for medication	Method of administration	Combined medication	Type of medications
Leehey et al. (38)	America	F	83	DM, hypertension, CKD	DM	Injection	–	–
Leehey et al. (38)	America	M	65	DM, hypertension, CKD	–	Injection	–	–
Halderman Kandarpa (21)	America	M	77	T2DM, hypertension, hyperlipidemia	T2DM	Oral	Metformin	–
Borkum et al. (22)	Canada	M	30+	DM, obesity, NICM	Weight loss, DM	Injection	Carvedilol, spironolactone, furosemide, ascorbic acid, metformin, rosuvastatin, sacubitril-valsart	BB, ARB, DIU
Chaiban et al. (23)	America	F	59	T2DM, hypothyroidism, breast cancer	T2DM	Injection	–	–
Kundu et al. (24)	America	F	57	Hypertension	Weight loss	Injection	Bumatenide, lisinopril, hydrochlorothiazide	ACEI, DIU
Ma et al. (25)	America	M	51	T2DM, hypertension, hyperlipidemia, NAFLD, CKD	T2DM	Oral	Pitavastatin, losartan, insulin	ARB
Hasan and Doroshow (26)	America	M	75	–	–	–	Metformin, ramipril, hydrochlorothiazide	ACEI, DIU
Herberth et al. (27)	America	F	55	ESRD, kidney transplantation	Weight loss	Injection	Tacrolimus, mycophenolate, prednisone	–
Patel et al. (28)	America	F	61	T2DM, heart transplantation, and CKD	T2DM	Injection	Tacrolimus	–
Barney et al. (29)	America	M	58	T2DM, HIV, hypertension	T2DM	Injection	–	–
Begum et al. (15)	America	F	68	Obesity, T2DM, hypertension, hyperlipidemia, coronary artery disease, hypothyroidism, CKD, gastroesophageal reflux disease	T2DM	Injection	Aspirin, atorvastatin, carvedilol, escitalopram, ferrous sulfate, furosemide, insulin, levothyroxine, lisinopril, morphine, pantoprazole, trazodone	BB, DIU, ACEI
Begum et al. (15)	America	F	49	Obesity, hypertension	Weight loss	Injection	Amlodipine, valsartan	CCB, ARB
Giofrè et al. (30)	Italy	F	29	Obesity,T2DM with microangiopathy microangiopathyhypertensive cardiomyopathy	–	Injection	Metoprolol, furosemide, nifedipine, doxazosina mesilato	BB, DIU, CCB
Kempster et al. (31)	America	F	61	T2DM, hypertension, hyperlipidemia	T2DM	–	–	–
Phillips et al. (32)	Australia	F	43	–	Weight loss	–	–	–
Tan et al. (33)	America	M	83	Obesity, hypertension, hyperlipidemia, CHF, CKD, hypothyroidism	Weight loss	–	–	–
Gu et al. (34)	China	F	53	T2DM, hypertension, hyperlipidemia	Weight loss, T2DM	Injection	Metformin, gliclazide, dapagliflozin, sacubitril/valsartan, lercanidipine, atorvastatin	ARB, CCB
Kovvuru et al. (35)	America	F	43	Abdominal liposuction	Weight loss,	Injection	–	–
Singhal et al. (36)	India	F	48	T2DM, hypertension, gastroesophageal reflux with a mild stricture and small hiatal hernia, and chronic constipation	T2DM	Injection	Metformin, lisinopril, hydrochlorothiazide, sucralfate, omeprazole, docusate	ACEI, DIU

DM, diabetes mellitus; CKD, chronic kidney disease; NICM, nonischemic cardiomyopathy; NAFLD, nonalcoholic fatty liver disease; ESRD, end stage kidney disease; NASH, nonalcoholic steatohepatitis; HIV, human immunodeficiency virus; CHF, congestive heart failure; BB, β-adrenergic receptor antagonists; ARB, angiotensin II receptor blocker; DIU, diuretic; ACEI, angiotensin-converting enzyme inhibitor; CCB, calcium channel blocker; F, female; M, male. “–”, not mentioned.

### 3.3 Clinical characteristics

The reported ADRs included acute kidney injury (AKI), CKD, acute renal failure (ARF), acute interstitial nephritis (AIN), focal segmental glomerulosclerosis (FSGS), minimal change disease (MCD), and nephrotic syndrome, with AKI being the most frequent. Among the 20 patients, 12 cases were diagnosed with kidney diseases based on renal biopsy findings, and the remaining eight cases were diagnosed through renal function assessments. Except for five cases with unknown dosages, semaglutide doses ranged from 0.25 to 2.0 mg per week, with 0.5 mg weekly being the most common. In addition, except for two patients whose time to ADR onset was unknown and one patient who developed ADR symptoms within 1 week, ADR onset occurred over several weeks in the remaining 17 patients, with the longest reported duration being 5 months. Clinical manifestations of the patients were diverse, including aggravated edema, decreased appetite, fatigue, altered mental state, oliguria, shortness of breath, reduced urine output, nausea, vomiting, diarrhea, jaundice, abdominal pain, progressive edema, fever, chills, and constipation. Among cases with available data, only one had a history of drug re-exposure. Treatment measures were clearly described in 17 patients. The physical conditions of 17 patients improved or recovered, while outcomes were not reported for two patients, and one patient did not show recovery. The detailed clinical characteristics are summarized in Table 2.

**TABLE 2:** The clinical characteristics of the 20 included cases.

References	ADR	Diagnosis	Dosage of ADR	Occurrence time	Clinical presentation	Drug re-exposure (Yes/No)	Intervention measures	Outcome
Leehey et al. (38)	AKI	Renal biopsy	0.5 mg weekly	5 months	Increasing leg edema	No	–	Unrecovered
Leehey et al. (38)	AKI	Kidney function	0.5 mg weekly	4 months	Decreased appetite and fatigue	No	–	Improved
Halderman Kandarpa (21)	AKI	Kidney function	–	–	Altered mental status, oliguric	No	Meformin discontinued; CRRT then transtioned to conventional hemodiaylsis	Recovered
Borkum et al. (22)	AKI	Renal biopsy	0.5 mg weekly	4 weeks	Malaise and increasing shortness of breath	No	Acute intermittent hemodialysis was initiated and subsequently continued for a week Prednisone 1 mg/kg and discharged on a tapering oral prednisone regimen	Recovered
Chaiban et al. (23)	ARF	Renal biopsy	0.25 mg weekly	4 weeks	Decreased urinary output nausea, vomiting	–	4 hemodialysis sessions over the course of 10 days	Recovered
Kundu et al. (24)	AKI	Kidney function	0.5 mg weekly	<1 month	Decreased urinary output, nausea, vomiting, generalized weakness	No	Antihvpertensives were discontinued, intravenous fluids	Recovered
Ma et al. (25)	KI	Renal biopsy	7 mg daily	5 months	Abdominal discomfort, newonset jaundice, nausea, vomiting, diarrhea, weakness, scleral icterus, dry oral mucosa	–	Hemodialysis, kidney transplantation	Recovered
Hasan and Doroshow (26)	AIN	Renal biopsy	–	4 months	Reduced urine output, nausea, vomiting, abdominal pain	No	Fluid resuscitation, intravenous methyl prednisone for 3 days followed by a prednisone taper; mycophenolate	–
Herberth et al. (27)	FSGS	Renal biopsy	2.0 mg weekly	2 months	Progressive edema, dyspnea	–	–	–
Patel et al. (28)	AKI	Renal biopsy	1.0 mg weekly	4 months	Decreased appetite, nausea, vomiting, diarrhea	No	Intravenous fluids	Improved
Barney et al. (29)	ARF	Kidney function	–	2 months	No	No	Intravenous fluids	Improved
Begum et al. (15)	AIN	Renal biopsy	0.25 mg weekly	3 weeks	Nausea, vomiting	Yes	A normal saline infusion; furosemide, lisinopril and semaglutide were discontinued; prednisone 60 mg daily which was gradually tapered over 3 months	Improved
Begum et al. (15)	FSGS, AIN with mild interstitial fibrosis and tubular atrophy	Renal biopsy	0.5 mg weekly	4 weeks	Bilateral pedal edema	No	0.5 mg/kg prednisone for 4 weeks, with dose-tapering by 10 mg every 2 weeks	Improved
Giofrè et al. (30)	ARF	Kidney function	0.5 mg weekly	3 weeks	Malaise, severe nausea, oliguric peripheral edema	No	Hydrated with 0.9% NaCl isotonic physiological solution in continuous infusion	Recovered
Kempster et al. (31)	AKI	Kidney function	–	2 months	Nausea, vomiting, diarrhea, poor oral intake, lethargic, tremulous	–	Hemodialysis, IV fluids, electrolyte repletion, rasburicase	Improved
Phillips et al. (32)	MCD	Renal biopsy	–	1 week	Lower extremity edema	No	6-weeks prednisone taper which was followed by tacrolimus 2 mg twice a day	Improved
Tan et al. (33)	Global glomerulos- clerosis	Renal biopsy	–	–	Dyspnea, bilateral leg swelling	–	Pulse solumedrol for 3 days and transitioned to a 3-months prednisone taper, received 2 doses of rituximab during the admission	Improved
Gu et al. (34)	AKI	Kidney function	1.0 mg weekly	–	Persistent nausea, vomiting, diarrhea	No	Metformin, dapagliflozin, sacubitril/valsartan were discontinued; fluid resuscitation, volume expansion, hemodialysis	Recovered
Kovvuru et al. (35)	Nephrotic syndrome	Renal biopsy	0.25 mg weekly	2 weeks	Bilateral lower extremity edema, foamy urine	No	Prednisone 60 mg po daily with a rapid taper to 10 mg daily by 4 weeks, tacrolimus 3 mg twice a day (4 months)	Recovered
Singhal et al. (36)	AKI	Kidney function	2.0 mg weekly	4 weeks	Nausea, non-bilious, non-bloody emesis, left lower quadrant abdominal pain, subjective fevers, chills, constipation	–	10 mg metoclopramide as needed every 6 h and maintenance fluid	Recovered

AKI, acute kidney injury; CKD, chronic kidney disease; ARF, acute renal failure; AIN, acute interstitial nephritis; FSGS, focal segmental glomerulosclerosis; MCD, minimal change disease; CRRT, continuous renal replacement therapy; “–”, not mentioned.

### 3.4 Laboratory test

Blood urea nitrogen levels were reported in 13 cases. With the exception of one case within the normal range, the remaining 13 cases showed values outside the normal range. Scr levels were available for 19 cases, of which 17 cases were above the normal range. eGFR was reported in eight cases, all of which were below the normal reference range. Among the nine cases reporting UPCR, eight showed elevated levels; moreover, proteinuria was reported in nine cases. Table 3 summarizes the kidney-related laboratory findings.

**TABLE 3:** Laboratory tests related to the kidneys of the 20 included cases.

References	BUN (mg/dl)	Scr (mg/dl)	eGFR (ml/min/1.73 m^2^)	UPCR (mg/g)	Proteinuria
Leehey et al. (38)	–	3.50	11	4900	3+
Leehey et al. (38)	–	–	22	1333	–
Halderman Kandarpa (21)	65	6.4	–	–	–
Borkum et al. (22)	165.53	12.86	–	91.1	Trace proteinuria
Chaiban et al. (23)	–	6.54	6 ml/min	–	–
Kundu et al. (24)	70	8.5	5.14	–	–
Ma et al. (25)	58	2.89	–	–	–
Hasan and Doroshow (26)	119	13.8	–	–	–
Herberth et al. (27)	–	1.2–1.5	–	3300 at 2 months 9000 at 4 months	16 g/24 h
Patel et al. (28)	83	12.5	–	1830	1+
Barney et al. (29)	55	5.94	–	–	2+
Begum et al. (15)	46	6.31	–	–	1+
Begum et al. (15)	–	1.6	–	11000	–
Giofrè et al. (30)	112	5.4	10	–	–
Kempster et al. (31)	135	14.2	3	–	–
Phillips et al. (32)	–	0.8	–	11010	3+
Tan et al. (33)	34	2.06	–	17200	4+
Gu et al. (34)	99.12	9.33	4.99	–	–
Kovvuru et al. (35)	10	0.8	–	11100	3+
Singhal et al. (36)	–	2.1	32 ml/min	–	–

BUN, blood urea nitrogen; Scr, serum creatinine; eGFR, estimated glomerular filtration rate; UPCR, urine protein-to-creatinine ratio.

### 3.5 Quality assessment

The quality assessment based on the JBI Case Report Checklist showed an overall low risk of bias in 20 cases included in the 18 studies. A total of 11 cases (55.00%, 11/20) fully demonstrated the key information. Among the remaining nine cases, 8 (40.00%, 8/20) cases lacked only one key information, whereas one (5%, 1/20) case did not mention three key elements. All included studies discussed the patients’ clinical conditions, diagnostic procedures, and adverse events. The detailed results of the quality assessment are presented in Table 4.

**TABLE 4:** Results of quality assessment.

References	Were the patient’s demographic characteristics clearly described?	Was the patient’s history clearly described and presented as a timeline?	Was the current clinical condition of the patient on presentation clearly described?	Were diagnostic tests or assessment methods and the results clearly described?	Was the intervention(s) or treatment procedure(s) clearly described?	Was the post-intervention clinical condition clearly described?	Were adverse events (harms) or unanticipated events identified and described?	Does the case report provide takeaway lessons?
Leehey et al. (38)	Yes	Yes	Yes	Yes	No	Yes	Yes	Yes
Leehey et al. (38)	Yes	Yes	Yes	Yes	No	Yes	Yes	Yes
Halderman Kandarpa (21)	Yes	No	Yes	Yes	Yes	Yes	Yes	Yes
Borkum et al. (22)	Yes	Yes	Yes	Yes	Yes	Yes	Yes	Yes
Chaiban et al. (23)	Yes	No	Yes	Yes	Yes	Yes	Yes	Yes
Kundu et al. (24)	Yes	Yes	Yes	Yes	Yes	Yes	Yes	Yes
Ma et al. (25)	Yes	Yes	Yes	Yes	Yes	Yes	Yes	No
Hasan and Doroshow (26)	Yes	Yes	Yes	Yes	Yes	No	Yes	Yes
Herberth et al. (27)	Yes	Yes	Yes	Yes	No	No	Yes	No
Patel et al. (28)	Yes	Yes	Yes	Yes	Yes	Yes	Yes	Yes
Barney et al. (29)	Yes	Yes	Yes	Yes	Yes	Yes	Yes	Yes
Begum et al. (15)	Yes	Yes	Yes	Yes	Yes	Yes	Yes	Yes
Begum et al. (15)	Yes	Yes	Yes	Yes	Yes	Yes	Yes	Yes
Giofrè et al. (30)	Yes	Yes	Yes	Yes	Yes	Yes	Yes	No
Kempster et al. (31)	Yes	Yes	Yes	Yes	Yes	Yes	Yes	No
Phillips et al. (32)	Yes	Yes	Yes	Yes	Yes	Yes	Yes	Yes
Tan et al. (33)	Yes	Yes	Yes	Yes	Yes	Yes	Yes	Yes
Gu et al. (34)	Yes	Yes	Yes	Yes	Yes	Yes	Yes	Yes
Kovvuru et al. (35)	Yes	Yes	Yes	Yes	Yes	Yes	Yes	Yes
Singhal et al. (36)	Yes	Yes	Yes	Yes	Yes	Yes	Yes	Yes

## 4 Discussion

Glucagon-like peptide-1 receptor agonists are widely used to treat T2DM and offer benefits in weight reduction, and improvements in cardiovascular and kidney outcomes, exhibiting an overall clinical significance in improving mortality (15). These drugs are expected to be more widely used in patients with and without kidney diseases and incorporated into an increasing number of drug combinations aimed at slowing the progression of diabetic nephropathy (22). However, there have been several case reports of AKI caused by GLP-1RAs in a cardiovascular outcome trial; however, the results of the meta-analysis have indicated that GLP-1RAs do not significantly increase the overall risk of AKI (37). In fact, since the widespread use of GLP-1Ras in 2015, the number of case reports of kidney diseases triggered by GLP-1RAs has increased rapidly. In 2021, Leehey et al. reported that patients with CKD treated with semaglutide developed biopsy-confirmed AKI and showed no improvement in renal function even after discontinuation of semaglutide (38). However, a clinical trial conducted on patients with T2D and CKD, aiming to explore the impact of semaglutide on CKD in patients with T2D, showed that semaglutide reduced the clinical risk of major renal outcomes (11). These conflicting findings on the effects of semaglutide on the kidneys highlight the need for a more comprehensive evaluation to characterize the clinical and biochemical features and treatment outcomes in patients with semaglutide-induced kidney damage. Therefore, we conducted a systematic review to comprehensively evaluate the case reports and case series related to kidney diseases associated with semaglutide use and included data of 20 cases derived from 18 published studies. The results indicate that the use of semaglutide may cause kidney damage, including severe renal complications with variable recovery in elderly patients with pre-existing kidney disease or metabolic syndrome, and individuals receiving concomitant medications.

It is crucial to identify the risk factors for semaglutide-associated renal adverse events to guide clinical monitoring of patients during the early stages of treatment. A systematic review of case reports involving GLP-1RA-related renal ADRs identified 23 cases, most of which occurred in patients with pre-existing kidney disease (39). Another study reported that a patient on chronic metformin treatment developed symptoms of AKI and lactic acidosis after initiation of semaglutide, suggesting that the high acute onset characteristic of the disease became more severe due to metformin toxicity, highlighting the risks associated with drug combinations of semaglutide (21). In the present systematic review, the majority of patients were older than 40 years. Although some patients used semaglutide for weight loss, except for one case with unknown history, nearly all patients had a history of underlying chronic conditions, including diabetes, hypertension, CKD, or metabolic disorders, and were receiving concomitant medications. A review of case reports on AIN and MCD associated with GLP-1 RAs have indicated that most patients were older and had a history of CKD, identifying potential risk factors such as CKD, advanced age, obesity, and concurrent use of AIN-inducing drugs (22). Our results are largely consistent with these observations, emphasizing the relevance of these factors in GLP-1 RA-associated renal injury. Notably, among the 20 cases of semaglutide-related kidney injury included in this study, at least 11 occurred in patients without a history of kidney disease.

Acute kidney injury has a high mortality rate and lacks effective treatment options. AKI may progress to CKD and eventually to end-stage renal failure (40). AIN is a significant contributor to AKI, and many cases of AKI with unknown etiology may actually correspond to AIN, particularly when non-invasive diagnostic tools are used instead of renal biopsy. AIN can be triggered by drugs, infections, autoimmune diseases, or idiopathic factors, with drug-induced AIN being the primary cause in many countries (41). A study conducted a comprehensive analysis of the data from the FAERS database related to the use of GLP-1 RAs and adverse renal events, and the results revealed that AKI was the most common condition (15). Our systematic review showed that the most common ADRs were AKI, ARF, and AIN. Twelve cases were diagnosed through renal biopsy, and the remaining eight cases were diagnosed based on renal function parameters. These ADRs occurred at doses within the range of standard clinically recommended doses, and most of the patients experienced these reactions only after 2–3 administrations. This suggests that clinicians should promptly monitor changes in renal function following the initiation of semaglutide, even at standard doses, to prevent the occurrence of AKI or even ARF. Furthermore, The clinical manifestations of the included patients were diverse, often accompanied by gastrointestinal symptoms such as loss of appetite, nausea, vomiting, diarrhea, abdominal pain, constipation, altered mental state, and shortness of breath, in addition to typical renal manifestations such as edema, oliguria, reduced urine output, and persistent edema, along with signs of infection such as fever and chills. This provides a diagnostic framework for clinicians, enabling them for timely diagnosis and identification of the associated causes.

Although pharmacological monitoring (serum drug concentration) was not reported in the included cases of renal ADRs such as blood drug concentration, and instances of drug re-exposure were limited, the symptoms and clinical outcomes were all related to the initiation and discontinuation of semaglutide use. For example, a case involving a 29-years-old woman from Italy, who had no history of CKD, showed a rapid decline in kidney function associated with the initiation and increase in semaglutide doses. Although the patient received other treatments, the dosage was maintained for at least 1 or 2 years. Notably, discontinuation of semaglutide resulted in a rapid and sustained improvement in kidney function (30), indicating semaglutide as the most likely causative factor. Management strategies reported across cases mainly focused on fluid replacement and administration of steroids. These treatment approaches led to clinical improvement or complete recovery. Although the exact mechanism underlying semaglutide-induced kidney damage or deterioration of kidney disease remains unclear, several mechanisms related to the effectiveness of the treatment have been proposed. First, gastrointestinal symptoms caused by semaglutide, such as nausea and vomiting, alongside the concurrent use of other medications such as angiotensin-converting enzyme inhibitors and diuretics, may lead to severe loss of body fluids and electrolytes, causing hypovolemia and insufficient renal perfusion. The early manifestation is prerenal AKI. If ischemia persists and worsens, it can lead to acute tubular necrosis (ATN), which is a typical pathological type of ischemic AKI (39). Second, as a peptide-based drug with potential immunogenicity, semaglutide may trigger systemic type 2 hypersensitivity reaction resembling drug-induced AIN. Responsiveness to steroid hormone treatment in some patients supports this hypothesis (15, 42). Additionally, genetic susceptibility may also play a role in such outcomes (25).

In summary, given the increasing number of case reports on GLP-1 ARs-induced renal injury (15), caution is required when prescribing these agents at clinical doses, particularly in patients with advanced CKD and insufficient kidney reserve. Special consideration should be given to the impact of adverse gastrointestinal reactions on volume status. Cases of renal damage in patients without CKD following semaglutide highlights the need for further investigation on the association of dehydration with the higher risk. Meanwhile, further research is needed to determine whether semaglutide exhibits greater immunogenicity compared with other drugs, and whether specific patients may benefit from other GLP-1RAs, as they have overall good health status. Large-scale, multi-center, prospective studies are required to determine risk factors, clinical manifestations, diagnostic approaches, and treatment plans. This study has few limitations. First, the number of included studies was limited, and all were case reports and case series involving individual patient data, making it unclear whether the results of this study can be applied to a broader population. Second, the absence of a control group in the included studies, making it impossible to infer causal relationships, may introduce potential biases. Furthermore, the absence of causality assessment (e.g., Naranjo or WHO-UMC) may weaken the conclusion. Finally, the preferential publication of positive or unusual cases may lead to publication bias.

## 5 Conclusion

Although semaglutide has been widely used due to its efficacy in weight reduction and glycemic control, this first systematic review of case series highlights that semaglutide-associated kidney damage is a major clinical concern. When prescribing semaglutide, particularly in elderly patients, with pre-existing kidney disease or metabolic syndrome, and individuals receiving concomitant medications, careful monitoring is essential. Clinicians should conduct regular follow-ups, including clinical biochemical parameters and assessment of renal function, and accordingly adjust the dosage of medication or modify the treatment regimen. Additionally, additional prospective studies with larger sample sizes and strict kidney assessment are needed to clarify the potential causal relationship, ultimately facilitating safe and effective use of semaglutide.

## Data Availability

The original contributions presented in this study are included in this article/Supplementary material, further inquiries can be directed to the corresponding authors.
