# Supplementary material for: A systematic review of semaglutide-associated kidney injury case reports
Source: Front Med (Lausanne). 2026 Jun 1;13:1793349. doi: 10.3389/fmed.2026.1793349 (PMC13265316; doi:10.3389/fmed.2026.1793349)
Supplement: Supplementary file 1 [file Table_1.docx]

**Supplementary Table S1.** The search queries used for each database and the search results

|  | Query | **Results (No.)**  **(2025.10.31)** |
| --- | --- | --- |
| **PubMed** | | |
| #1 | semaglutide[Title/Abstract] | 3,598 |
| #2 | (case report) OR (case presentation) OR (case summary) OR (case description) OR (case discussion) | [3,099,3](https://pubmed.ncbi.nlm.nih.gov/?term=((((case+report)+OR+(case+presentation))+OR+(case+summary))+OR+(case+description))+OR+(case+discussion)&sort=date)84 |
| #3 | #1 AND #2 | **253** |
| **Embase** | | |
| #1 | semaglutide:ti,ab,kw | 6,612 |
| #2 | 'case report' OR 'case presentation' OR 'case summary' OR 'case description' OR 'case discussion' | 3,339,768 |
| #3 | #1 AND #2 | **425** |
| **Cochrane Library** | | |
| #1 | (semaglutide):ti,ab,kw | 1,700 |
| #2 | (case report OR case presentation OR case summary OR case description OR case discussion ) | 85,733 |
| #3 | #1 AND #2 | 21（18个临床试验） |
| **Web of Science** | |  |
| #1 | semaglutide | 5.936 |
| #2 | (case report) OR (case presentation) OR (case summary) OR (case description) OR (case discussion) | 2,824,018 |
| #3 | #1 AND #2 | **282** |
